# FBP1 Is Associated with Attenuated Mitochondrial Injury in Renal Tubular Epithelial Cells of Diabetic Kidney Disease via Modulation of Lactate Metabolism

**DOI:** 10.3390/ijms27135906

**Published:** 2026-06-30

**Authors:** Siyi Rao, Mengjie Weng, Yongjie Zhuo, Jiaqun Lin, Danyu You, Jiong Cui, Yi Chen, Xiaohong Zhang, Jianxin Wan

**Affiliations:** 1 Department of Nephrology, Blood Purification Research Center, The First Affiliated Hospital, Fujian Medical University, Fuzhou 350005, China; yy03715@fjtcm.edu.cn (S.R.);; 2 Fujian Clinical Research Center for Metabolic Chronic Kidney Disease, The First Affiliated Hospital, Fujian Medical University, Fuzhou 350005, China; 3 Department of Nephrology, National Regional Medical Center, Binhai Campus of the First Affiliated Hospital, Fujian Medical University, Fuzhou 350212, China

**Keywords:** diabetic kidney disease, lactate metabolism regulation, mitochondrial injury, Fructose-1,6-bisphosphatase 1, lactate dehydrogenase B

## Abstract

The role of gluconeogenesis in kidney disease has increasingly drawn attention. Fructose-1,6-bisphosphatase 1 (FBP1) is a key rate-limiting enzyme in gluconeogenesis that suppresses glycolysis and reduces lactate production. In this study, we first analyzed public transcriptomic datasets of diabetic kidney disease (DKD) and validated the findings in 24-week-old BKS-db mice and in high-glucose-induced human renal tubular epithelial (HK-2) cells. We further constructed tubular-specific FBP1 overexpression/knockdown mouse models via adeno-associated virus serotype 9 (AAV-9) and combined pharmacological inhibition of lactate dehydrogenase B (LDHB) to dissect the underlying mechanism. Analysis of public clinical transcriptomic datasets showed that renal tubular FBP1 expression was positively correlated with estimated glomerular filtration rate (eGFR). In vivo, tubular-specific FBP1 overexpression in BKS-db mice reduced 24-h urinary protein and decreased renal lactate accumulation (*p* < 0.05) compared with diabetic controls. In vitro, high glucose-induced lactate elevation in HK-2 cells was reversed by FBP1 overexpression, while co-treatment with an LDHB inhibitor abolished this protective effect. Our findings suggest that FBP1 represents a potential experimental therapeutic target associated with alleviation of renal lactic acid accumulation and mitochondrial injury in preclinical DKD models.

## 1. Introduction

Diabetic kidney disease is the most common complication of diabetes, with an incidence rate of approximately 30% to 50% among diabetic patients. Currently, DKD has become the primary cause of end-stage renal disease (ESRD) [1,2]. The development of DKD is driven by a combination of hemodynamic alterations, inflammatory responses, metabolic disturbances, and other contributing factors. In recent years, the interactions between glucose metabolism pathways and mitochondrial function, as well as the role of the Warburg effect in the progression of DKD, have attracted increasing attention.

The kidneys are the second most energy-consuming organ in the body after the heart (400 kcal/kg of tissue per day) [3], and energy homeostasis is crucial for maintaining their function. Metabolic reprogramming of the kidneys in response to high glucose involves multiple mechanisms. Friederich et al. [4] found that sustained hyperglycemia induces an abnormally high proton gradient across the entire mitochondrial membrane, leading to excessive production of reactive oxygen and nitrogen species, thereby promoting mitochondrial dysfunction. Additionally, in early DKD models, increased renal blood flow and glomerular filtration rate may lead to ischemic kidney damage. Therefore, early DKD kidneys may exhibit “hypoxia”-related manifestations [5]. Hypoxia is a definitive driver of metabolic shifts from mitochondrial oxidative phosphorylation to anaerobic fermentation.

Gluconeogenesis is the process by which glucose is synthesized from non-glucose precursors. Previously, the liver was considered the primary organ responsible for systemic gluconeogenesis. However, recent studies have shown that under stress conditions such as acidosis, fasting, or high-glucose stimulation, the kidneys can account for approximately 40–50% of endogenous gluconeogenesis throughout the body [6]. The capacity for gluconeogenesis influences the interconversion of glucose, lactate, and pyruvate in the kidneys. When gluconeogenesis is reduced, lactate clearance decreases, leading to lactate accumulation at both local and systemic levels [7]. The concept of the pyruvate-lactate metabolic axis was first proposed by Ahmad A. Cluntun et al. in studies on myocardial cell metabolism in heart failure [8]. Interestingly, as in renal tubular epithelial cells, researchers suggest that myocardial cell metabolism is flexible and dynamic. Fatty Acid Oxidation (FAO) is also a primary source of ATP production in cardiomyocytes. During HF, the coupled pyruvate oxidation associated with FAO is disrupted, preventing pyruvate from entering the mitochondria for the Tricarboxylic Acid (TCA) Cycle. Instead, it is metabolized in the cytoplasm, with its metabolic products converted to lactate. Lactate-centered energy metabolism has emerged as a prominent focus in nephrology research [9]. Beyond its classical identity as a glycolytic end-product, lactic acid functions as a pivotal signaling metabolite that orchestrates diverse metabolic pathways and biological processes [10].

The expression patterns and functional roles of genes involved in glucose metabolism in DKD remain incompletely characterized. In this study, we conducted in silico analyses using two publicly available renal tubule transcriptomic datasets from the GEO database (GSE30529 and GSE104945). After removing batch effects and standardizing the data, we integrated weighted gene co-expression network analysis, differential expression analysis, and functional enrichment analysis to systematically evaluate glucose metabolism-related genes in normal and DKD renal tissues. We identified FBP1, a rate-limiting enzyme in the gluconeogenesis pathway, as a significantly downregulated gene in DKD patients, with expression levels showing a significant positive correlation with eGFR.

Current research on FBP1 primarily focuses on tumor-related studies. Bo Li et al. [11] found that FBP1 expression was universally absent in 600 patients with renal clear cell carcinoma and confirmed that FBP1 inhibits tumor progression by suppressing glycolytic flux in renal tubular epithelial cells. Research on FBP1 in kidney diseases is limited. Under normal physiological conditions, renal tubular epithelial cells contain abundant mitochondria and primarily rely on fatty acids as their primary fuel, generating energy via mitochondrial oxidative phosphorylation (Oxphos) [12]. When kidney damage occurs, renal tubular energy metabolism shifts to aerobic glycolysis for energy production [13]. In the renal cortex of BKS-db diabetic mice, studies have shown increased metabolic flux in glycolysis, FAO, and the TCA cycle [14]. We hypothesize that FBP1 may be involved in the progression of DKD potentially through modulating tubular energy metabolism phenotypes. In this study, we established DKD mouse models with tubular FBP1 overexpression and knockdown, as well as a high-glucose-induced tubular epithelial cell injury model. We explored phenotypic and mechanistic aspects, including changes in energy metabolism and mitochondrial damage, to provide insights into identifying new therapeutic targets for DKD.

## 2. Results

### 2.1. Pathway Enrichment Analysis of Renal Tubule-Associated Genes in DKD Subjects

**Figure 1:**
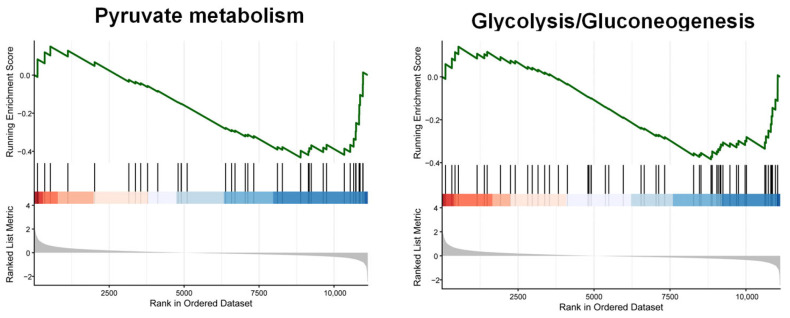
GSEA enrichment analysis results.

Gene Set Enrichment Analysis (GSEA) was performed on all background genes using the ClusterProfiler R package (4.5.2). Pathways with a normalized enrichment score (|NES| > 1) and false discovery rate-adjusted *p* < 0.05 were defined as significantly enriched. Among the 347 pathways identified, genes were significantly depleted in pyruvate metabolism (NES = −1.500, *p* = 0.027) and glycolysis/gluconeogenesis (NES = −1.436, *p* = 0.038) (Figure 1).

### 2.2. Weighted Gene Co-Expression Network Analysis and Core Module Identification

**Figure 2:**
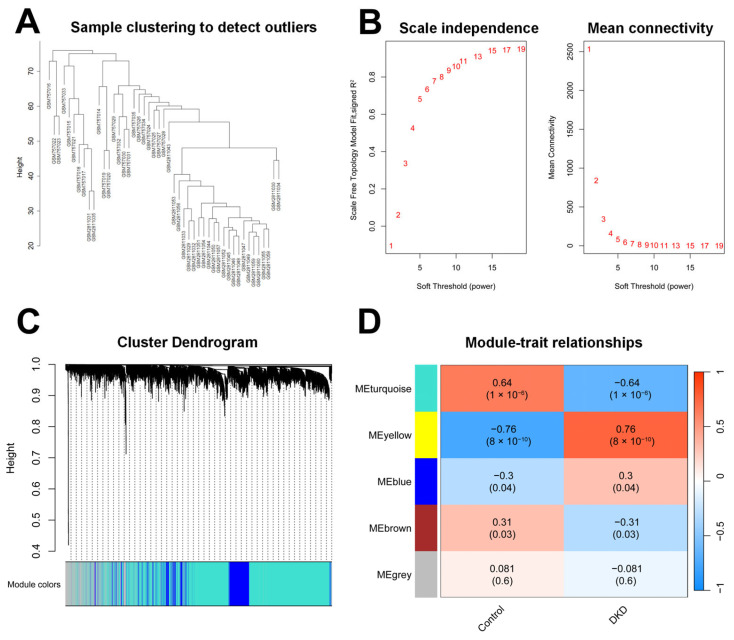
WGCNA was used to identify module genes associated with diabetic nephropathy tubules. (**A**) Sample clustering diagram. (**B**) Soft threshold screening. (**C**) Cluster analysis diagram. (**D**) Correlation analysis between gene modules and risk models and clinical characteristics.

The sample clustering diagram (Figure 2A) suggested no obvious outliers in the dataset. Weighted Gene Co-expression Network Analysis (WGCNA) was performed using the R package (4.5.2) of the same name. A soft threshold of 10 was selected to construct the scale-free network, and the adjacency matrix and topological overlap matrix (TOM) were subsequently calculated (Figure 2B). We then calculated the module feature genes representing the overall gene expression levels of each module; these were clustered based on their correlations (Figure 2C). We analyzed the correlation of each feature gene with the phenotype (DKD or control samples) and selected the two modules most significantly correlated with DKD (Figure 2D), namely the turquoise module (cor = −0.64, *p* = 1 × 10^−6^) and the yellow module (cor = 0.76, *p* = 8 × 10^−10^).

### 2.3. Acquisition of Core Genes Related to DKD Renal Tubules

**Figure 3:**
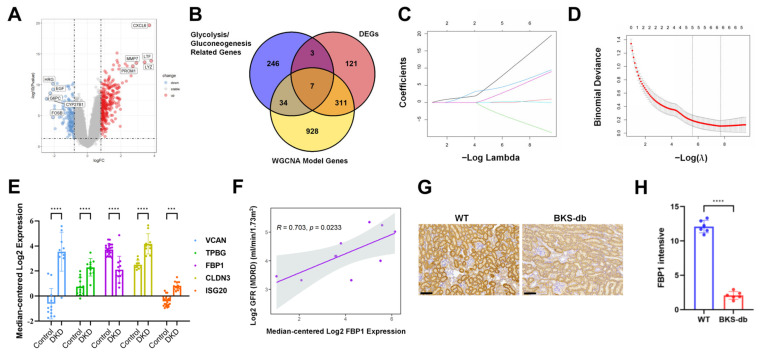
Hub gene acquisition and identification. (**A**) Volcano plot showing differentially expressed genes between the two groups. Differentially expressed genes are defined as |logFC| > 0.8 and *p*-value < 0.05. (**B**) Venn diagram showing the intersection of glycolysis/gluconeogenesis-related genes, differentially expressed genes, and module genes screened by WGCNA. (**C**,**D**) Lasso regression model construction. (**E**) Validation of key gene expression using the Nephrseq V5 dataset. (**F**) Correlation analysis between FBP1 and eGFR. (**G**) Representative images of FBP1 immunohistochemical staining in kidney tissue sections from control (WT) and BKS-db mice. Bar: 100 μm. (**H**) Statistical analysis of immunohistochemical staining. *n* = 6. *** *p* < 0.001, **** *p* < 0.0001.

A total of 442 DEGs were identified in the dataset, including 151 upregulated genes and 291 downregulated genes. The top five upregulated DEGs and the top five downregulated DEGs in the two datasets were identified by selecting columns with log-fold changes in ascending order from the results of analysis of variance (ANOVA) and displayed in a volcano plot (Figure 3A). We intersected sugar metabolism-related genes with module genes and differentially expressed genes obtained from WGCNA to obtain seven intersection genes (Figure 3B). Further screening using LASSO regression identified five core genes (*CLDN3*, *ISG20*, *TPBG*, *VCAN*, *FBP1*) (Figure 3C,D).

Nephrseq v5 showed that, compared with the control group, the mRNA expression levels of *CLDN3*, *ISG20*, *TPBG*, and *VCAN* were elevated in the kidneys of DKD patients, while the mRNA level of *FBP1* was downregulated (Figure 3E). *FBP1* was identified as the sole significantly downregulated gene within the dataset, rendering it a primary focus of subsequent investigation. Analysis of gene and eGFR data from Nephrseq V5 revealed that FBP1 mRNA expression levels in the renal tubules were positively correlated with eGFR in DKD patients (R = 0.703, *p* = 0.023), suggesting that regulating FBP1 expression in the kidney may help slow the progression of DKD (Figure 3F).

We further validated this conclusion in 24-week-old BKS-db mice. Immunohistochemical results indicated that FBP1 expression was primarily concentrated in the renal tubules, and compared with WT mice, FBP1 expression was significantly downregulated in BKS-db mice (*p* < 0.0001) (Figure 3G,H).

### 2.4. In Vivo Validation of FBP1 Overexpression in Animals

To investigate the experimental efficacy of enhancing renal tubular FBP1 expression in vivo, we constructed a tubular-specific FBP1 overexpression mouse model by tail vein injection of AAV9 carrying the FBP1 coding sequence under the Ksp-cadherin promoter. Age-matched wild-type (WT) mice, untreated BKS-db diabetic mice, and BKS-db mice injected with empty AAV9 vector were used as controls. Metabolic phenotypes, renal function, and tubular injury parameters were evaluated at 24 weeks of age to assess the impact of FBP1 overexpression on DKD progression.

**Figure 4:**
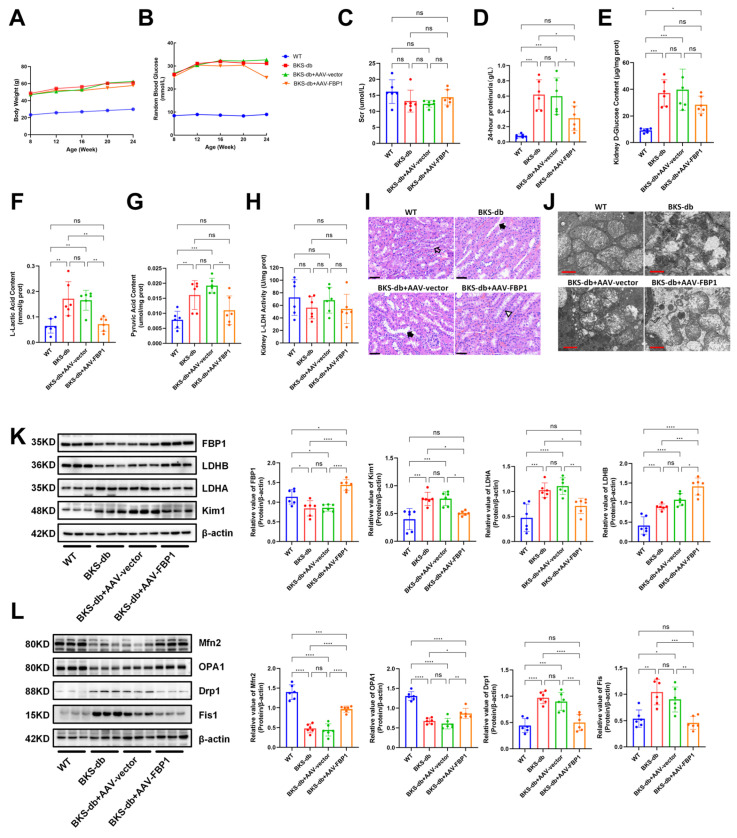
In vivo experimental results of the FBP1 overexpression animal model. (**A**) Changes in body weight (*n* = 6). (**B**) Standardized blood glucose levels (*n* = 6). (**C**) Comparison of serum creatinine levels. Note: Although measurements were taken weekly, statistical analyses were conducted as independent cross-sectional comparisons at each time point. (**D**) Comparison of 24-h urine protein levels. (**E**) Comparison of glucose content in the kidneys (*n* = 6). (**F**) Comparison of renal lactate levels (*n* = 6). (**G**) Comparison of renal pyruvate levels (*n* = 6). (**H**) Comparison of renal L-LDH activity (*n* = 6). (**I**) Renal HE staining observations (40×, Bar: 50 μm) (*n* = 6). Hollow arrows indicate intact tubular structure in the WT group, solid black arrows indicate brush border detachment, tubular lumen dilation and atrophy in the BKS-db and Vector groups, and hollow triangles indicate attenuated tubular damage in the FBP1 overexpression group. (**J**) Electron microscopic observations of mitochondrial morphology in renal tubules (10,000×, Bar: 1.0 μm) (*n* = 6). (**K**) Protein expression levels of FBP1, lactate dehydrogenases (LDHA/LDHB) and renal injury marker Kim1 in Expression of mitochondrial fusion and fission proteins in the kidneys of mice kidney tissues (*n* = 6). (**L**) Expression of mitochondrial fusion and fission proteins in the kidneys of mice (*n* = 6). WT: wild-type control mice; BKS-db: diabetic BKS-db mice; BKS-db+AAV-vector: BKS-db mice injected with empty AAV9 vector via tail vein; BKS-db+AAV-FBP1: BKS-db mice injected with AAV9 overexpressing FBP1 via tail vein. Data are presented as mean ± SD; *n* = 6 per group. Statistical comparisons were performed by one-way ANOVA. * *p* < 0.05, ** *p* < 0.01, *** *p* < 0.001, **** *p* < 0.0001, ns, no statistically significant difference.

During the experiment, we monitored changes in blood glucose and body weight in mice every 4 weeks. We found that the body weight changes in the FBP1 overexpression group were similar to those in the BKS-db group and vector group, both of which were significantly higher than the WT group. However, blood glucose levels in the FBP1 overexpression group began to decrease from week 20 onwards (Figure 4A,B). There were no significant differences in serum creatinine levels among the WT, BKS-db, empty vector, and FBP1 overexpression groups. However, compared with the BKS-db and empty vector groups, the 24-h urine protein levels in the FBP1 overexpression group were significantly reduced (*p* < 0.05) (Figure 4C,D).

Analysis of renal tissue glucose metabolism-related markers revealed no significant differences in renal glucose levels among the BKS-db, empty vector, and FBP1 overexpression groups (*p* > 0.05) (Figure 4E). However, compared with the BKS-db and empty vector groups, FBP1 overexpression significantly reduced renal lactate levels (*p* < 0.01) (Figure 4F). Compared with the empty vector group, the FBP1 overexpression group showed a significant decrease in renal pyruvate content (*p* < 0.01) (Figure 4G). There were no significant differences in L-LDH activity among the WT, BKS-db, empty vector, and FBP1 overexpression groups (Figure 4H).

HE staining results showed that compared with WT mice, BKS-db and empty vector groups exhibited more pronounced tubular epithelial cell or brush border detachment accompanied by tubular atrophy, while the FBP1 overexpression group exhibited milder tubular damage (Figure 4I). Compared with WT mice, BKS-db and BKS-db+AAV-vector mice exhibited significantly elevated levels of the renal injury marker Kim1, increased LDHA levels, and decreased LDHB levels. Overexpression of FBP1 decreased LDHA expression, and increased LDHB expression (Figure 4K).

Electron microscopy revealed that in the BKS-db and empty vector groups, the cell membranes were locally damaged, mitochondria were mildly swollen, the matrix was uneven, cristae were mostly reduced or absent, and some mitochondria had damaged membranes, broken cristae, and localized myeloid changes. Overexpression of FBP1 partially corrected the aforementioned cellular damage (Figure 4J). Mitochondrial-related protein assays also showed similar results, with overexpression of FBP1 increasing the expression levels of mitochondrial fusion proteins Mfn2 and OPA1 in tissues while decreasing the expression levels of mitochondrial fission proteins Fis and Drp1 (Figure 4L).

### 2.5. In Vivo Validation of FBP1 Knockdown

**Figure 5:**
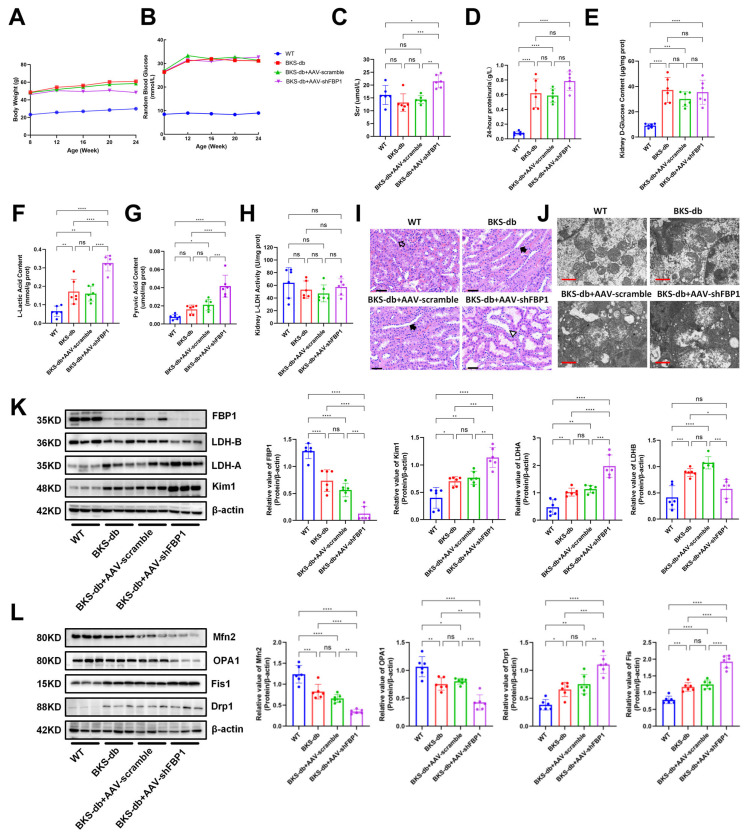
In vivo experimental results of the FBP1 knockdown animal model. (**A**) Changes in body weight (*n* = 6). (**B**) Standardized blood glucose levels (*n* = 6). Note: Although measurements were taken weekly, statistical analyses were conducted as independent cross-sectional comparisons at each time point. (**C**) Comparison of serum creatinine levels. (**D**) Comparison of 24-h urine protein levels. (**E**) Comparison of glucose content in the kidneys (*n* = 6). (**F**) Comparison of renal lactate levels (*n* = 6). (**G**) Comparison of renal pyruvate levels (*n* = 6). (**H**) Comparison of renal L-LDH activity (*n* = 6). (**I**) Renal HE staining observations (40×, Bar: 50 μm) (*n* = 6). Hollow arrows indicate intact tubular structure in the WT group, solid black arrows indicate brush border detachment, tubular lumen dilation and atrophy in the BKS-db and Vector groups, and hollow triangles indicate that the damage to the tubular structure was further exacerbated in the FBP1 knockdown group. (**J**) Electron microscopic observations of mitochondrial morphology in renal tubules (10,000×, Bar: 1.0 μm) (*n* = 6). (**K**) Protein expression levels of FBP1, lactate dehydrogenases (LDHA/LDHB) and renal injury marker Kim1 in Expression of mitochondrial fusion and fission proteins in the kidneys of mice kidney tissues (*n* = 6). (**L**) Expression of mitochondrial fusion and fission proteins in the kidneys of mice (*n* = 6). WT: wild-type control mice; BKS-db: diabetic BKS-db mice; BKS-db+AAV-vector: BKS-db mice injected with empty AAV9 vector via tail vein; BKS-db+AAV-FBP1: BKS-db mice injected with AAV9 overexpressing FBP1 via tail vein. Data are presented as mean ± SD; *n* = 6 per group. Statistical comparisons were performed by one-way ANOVA. * *p* < 0.05, ** *p* < 0.01, *** *p* < 0.001, **** *p* < 0.0001, ns, no statistically significant difference.

Compared to the BKS-db and empty vector groups, body weight showed a progressive decrease, but blood glucose levels remained persistently elevated (Figure 5A,B). Serum creatinine levels were significantly elevated, and 24-h urine protein levels remained at elevated levels (Figure 5C,D).

Glucose levels in the kidneys remained elevated, but renal lactate and pyruvate levels were significantly increased (Figure 5E–G). There were no significant differences in L-LDH activity among the WT, BKS-db, empty vector, and FBP1 knockdown groups (Figure 5H).

HE staining results indicated that compared with BKS-db and empty vector group mice, FBP1 knockdown resulted in more pronounced tubular epithelial cell or brush border detachment and tubular atrophy (Figure 5I), with further increased Kim1 expression, elevated LDHA expression, and decreased LDHB expression (Figure 5J).

Electron microscopy results showed that, compared with BKS-db and empty vector groups, FBP1 knockdown group mice exhibited extreme mitochondrial matrix heterogeneity, marked mitochondrial swelling, and most mitochondria were damaged under the microscope, with cristae rupture and localized cristae myeloidization (Figure 5I). Mfn2 and OPA1 expression further decreased, while Drp1 and Fis expression further increased (Figure 5K).

### 2.6. In Vitro Validation of HK-2 Cells Overexpressing FBP1

**Figure 6:**
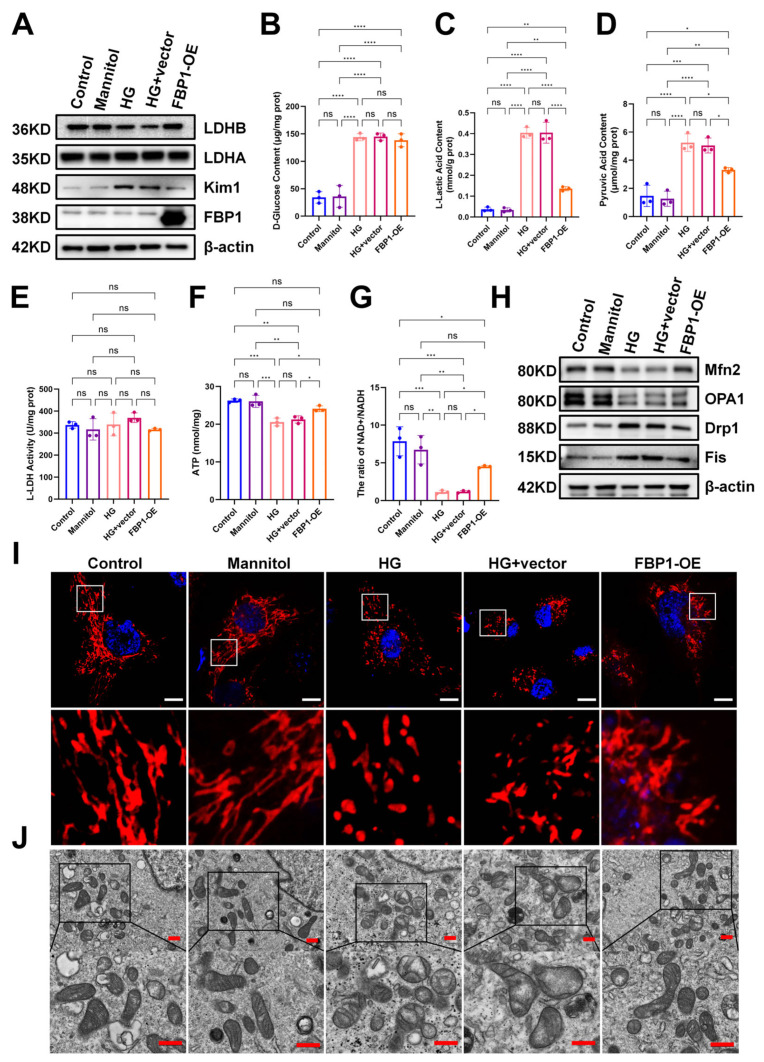
In vitro experimental results of HK-2 cells overexpressing FBP1. (**A**) Expression levels of lactate dehydrogenase, FBP1, and Kim1 (*n* = 3). (**B**) Comparison of glucose content (**C**) Comparison of lactate content (*n* = 3). (**D**) Comparison of pyruvate content (*n* = 3). (**E**) Expression levels of mitochondrial fusion and fission proteins (*n* = 3). (**F**) Comparison of L-LDH activity (*n* = 3). (**G**) Comparison of ATP content (*n* = 3). (**H**) Comparison of NAD+/NADH ratios (*n* = 3). (**I**) Mitochondrial morphology observed via MitoTracker staining (*n* = 3). Bar: 5 μm. (**J**) Mitochondrial morphology observed via electron microscopy (*n* = 3). Bar: 5 μm. Control: HK-2 cells cultured in normal glucose; Mannitol: HK-2 cells treated with 5 mM mannitol as osmotic control; HG: HK-2 cells treated with 30 mM high glucose; HG+vector: HG-treated cells transduced with empty lentiviral vector; FBP1-OE: HG-treated cells transduced with FBP1-overexpressing lentivirus. Data are mean ± SD; *n* = 3 independent biological replicates. Statistical comparisons were performed by one-way ANOVA. * *p* < 0.05, ** *p* < 0.01, *** *p* < 0.001, **** *p* < 0.0001, ns, no statistically significant difference.

We observed that FBP1 is primarily expressed in the renal tubule region. In the in vitro experimental section, we utilized lentivirus to construct FBP1 overexpression and knockdown models. The results showed that high glucose significantly increased Kim1 expression levels and decreased LDHB expression. However, in the HK-2 cell model, we noted that LDHA expression levels were significantly high, so no significant differences were observed between groups. Overexpression of FBP1 led to a decrease in Kim1 expression and an increase in LDHB expression (Figure 6A, Figure S1).

Regarding glucose metabolism indicators, compared with the high-glucose and empty vector groups, there were no significant differences in glucose levels in FBP1-OE group cells. However, high glucose levels led to increased cellular lactate and pyruvate levels, which significantly decreased after FBP1 overexpression (*p* < 0.05). No significant differences were observed among the groups in L-LDH activity (Figure 6B–E). In terms of energy metabolism, ATP and NAD+/NADH levels in HK-2 cells significantly decreased after high-glucose intervention, and overexpression of FBP1 increased cellular ATP and NAD+/NADH levels (Figure 6F,G).

Mitochondrial damage was consistent with in vivo experimental results. Under high-glucose induction, mitochondrial fusion protein levels in HK-2 cells significantly decreased, while fission protein expression significantly increased. Overexpression of FBP1 partially reversed the aforementioned protein expression patterns (Figure 6H, Figure S2). Additionally, Mitotracker staining results showed that compared to the high-glucose group, the FBP1-OE group exhibited reduced mitochondrial fragmentation and restored network-like structures. Under electron microscopy, we observed mild swelling of mitochondria, uneven matrix, reduced cristae in some mitochondria, and local myeloid changes in cristae in high-glucose-treated cells. Overexpression of FBP1 alleviated these mitochondrial damage manifestations (Figure 6I,J).

### 2.7. In Vitro Validation of FBP1 Knockdown in HK-2 Cells

**Figure 7:**
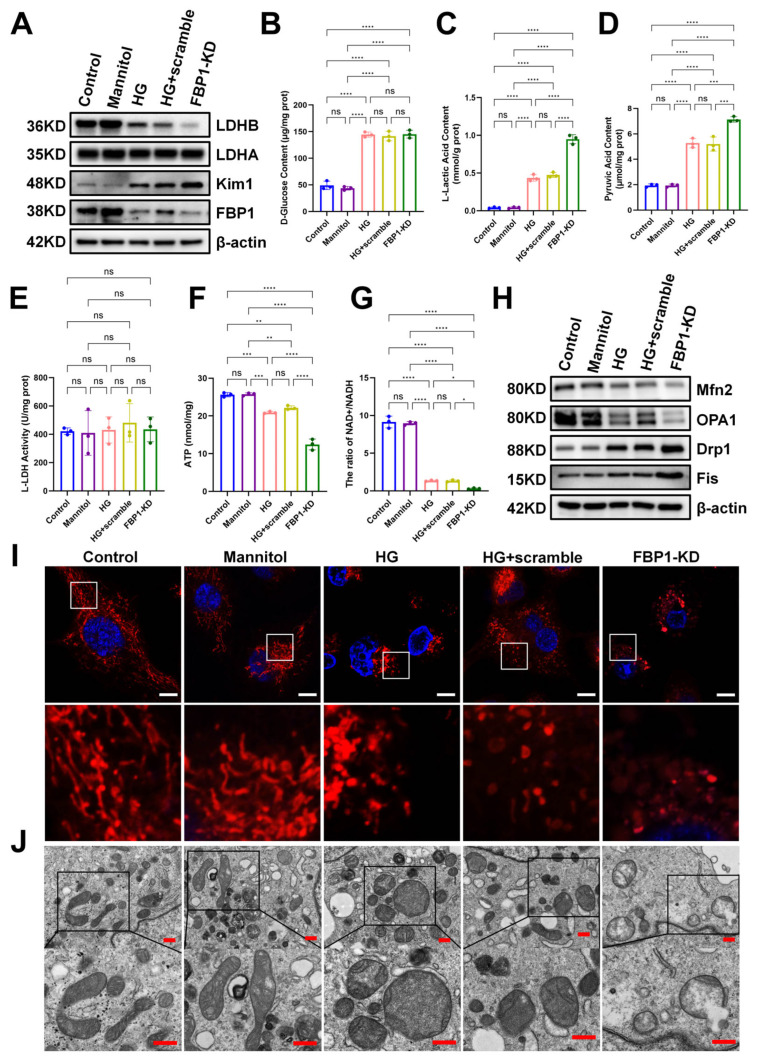
In vitro experimental results of HK-2 cells with FBP1 knockdown. (**A**) Expression levels of lactate dehydrogenase, FBP1, and Kim1 (*n* = 3). (**B**) Comparison of glucose content (*n* = 3). (**C**) Comparison of lactate content (*n* = 3). (**D**) Comparison of pyruvate content (*n* = 3). (**E**) Expression levels of mitochondrial fusion and fission proteins (*n* = 3). (**F**) Comparison of L-LDH activity (*n* = 3). (**G**) Comparison of ATP content (*n* = 3). (**H**) Comparison of NAD+/NADH ratios (*n* = 3). (**I**) Mitochondrial morphology observed via MitoTracker staining (*n* = 3). Bar: 5 μm. (**J**) Mitochondrial morphology observed via electron microscopy (*n* = 3). Bar: 5 μm. Control: HK-2 cells cultured in normal glucose; Mannitol: HK-2 cells treated with 5 mM mannitol as osmotic control; HG: HK-2 cells treated with 30 mM high glucose. Data are mean ± SD; *n* = 3 independent biological replicates. Statistical comparisons were performed by one-way ANOVA. * *p* < 0.05, ** *p* < 0.01, *** *p* < 0.001, **** *p* < 0.0001, ns, no statistically significant difference.

Knockdown of FBP1 further increased Kim1 expression and decreased LDHB expression in HK-2 cells induced by high glucose (Figure 7A, Figure S3). Compared with the high glucose and empty vector groups, glucose content in FBP1-KD cells did not show significant changes, but lactate and pyruvate content further increased (*p* < 0.05), and L-LDH activity did not show significant statistical differences (Figure 7B–E). ATP and NAD+/NADH levels in HK-2 cells after high-glucose intervention further decreased after FBP1 knockdown (*p* < 0.05) (Figure 7F,G).

Compared with the high-glucose and empty vector groups, Mfn2 and OPA1 expression further decreased, while Drp1 and Fis expression further increased in the FBP1-KD group (Figure 7H, Figure S4). MitoTracker staining results showed more severe mitochondrial fragmentation in the FBP1-KD group, with rod-shaped mitochondrial structures almost disappearing and no obvious reticulum-like structures observed. Under electron microscopy, mitochondrial damage was more pronounced in the FBP1-KD group, with mitochondria exhibiting severe swelling, most mitochondrial membranes ruptured, and mitochondrial cristae almost disappeared, presenting a myeloid-like appearance (Figure 7I,J).

### 2.8. Effects of LDHB Inhibitors on HK-2 Cells

**Figure 8:**
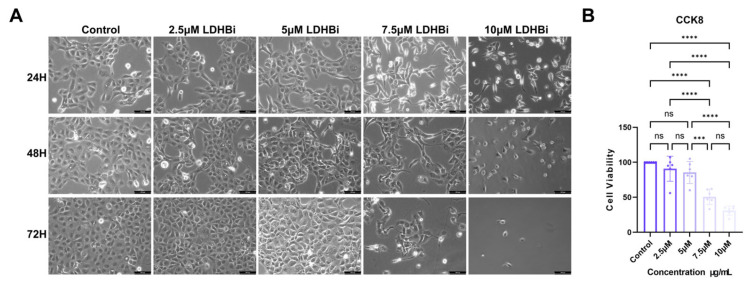
Effects of different LDHB inhibitor intervention concentrations and intervention time on HK-2 cell morphology. (**A**) Observation of the morphology of HK-2 cells under a light microscope (*n* = 3). Bar: 100 μm. (**B**) Dose-dependent cytotoxicity of LDHB inhibitor on HK-2 cells. *** *p* < 0.001, **** *p* < 0.001, ns, no statistically significant difference (*n* = 6).

HK-2 cells were treated with LDHB inhibitors at concentrations of 2.5 μM, 5 μM, 7.5 μM, and 10 μM for 24, 48, and 72 h, and morphological changes in HK-2 cells were observed under a light microscope. Compared with the Control group, after 72 h of treatment, there were no significant morphological changes in HK-2 cells in the 2.5 μM LDHBi group, the 5 μM LDHBi group exhibited poor cell morphology, with some cells appearing elongated and showing a few pseudopodia, and the 7.5 μM and 10 μM LDHBi group showed significant morphological changes, with the cobblestone structure almost completely lost. The cell viability of each group was detected using CCK8 after 72 h of LDHBi intervention. As shown in the bar graph, cell viability remained relatively unchanged (no significant difference, ns) between the control group and the 2.5 μM, 5 μM treatment group. At 5 μM LDHBi, no significant reduction in cell viability was observed, while marked morphological alterations occurred; higher concentrations (7.5 and 10 μM) induced dose-dependent viability loss. (Figure 8).

### 2.9. The Role of LDHB Inhibitors in Cell Experiments with FBP1 Overexpression

**Figure 9:**
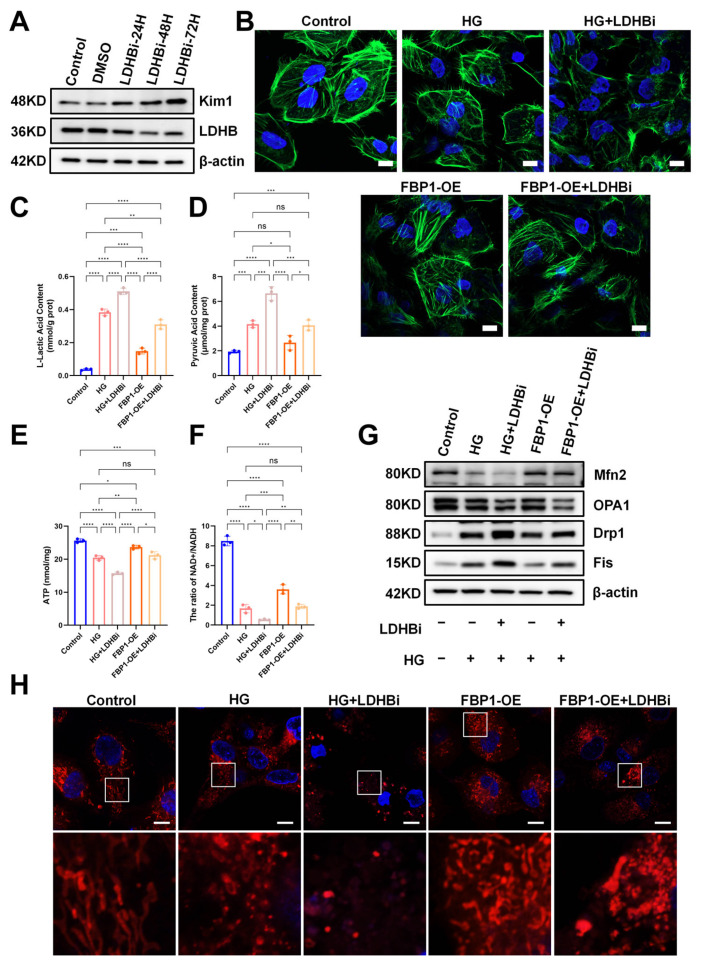
(**A**) Effects of different LDHB inhibitors on LDHB and Kim1 expression in HK-2 cells at different intervention times (*n* = 3). (**B**) Effects of LDHB inhibitors and FBP1 on the cytoskeleton of HK-2 cells (*n* = 3). Bar: 5 μm. (**C**,**D**) Effects of LDHB inhibitors on lactate levels in HK-2 cells overexpressing FBP1 under high-glucose conditions (*n* = 3). (**E**,**F**) Effects of LDHB inhibitors on ATP and NAD+/NADH levels in high-glucose-treated HK-2 cells overexpressing FBP1 (*n* = 3). (**G**) Effects of LDHB inhibitors and FBP1 on the expression of mitochondrial-related proteins in HK-2 cells (*n* = 3). (**H**) Mitotracker staining to observe mitochondrial morphology in each group of cells (*n* = 3). Bar: 10 μm. Control: control group HK-2 cells. HG: High-glucose-treated HK-2 cell group. HG+LDHBi: High-glucose and LDHB inhibitor-treated group. FBP1-OE: High-glucose-treated FBP1-overexpressing HK-2 cell group. FBP1-OE+LDHBi: High-glucose-treated FBP1-overexpressing + LDHB inhibitor group. * *p* < 0.05, ** *p* < 0.01, *** *p* < 0.001, **** *p* < 0.0001, ns, no statistically significant difference.

We observed the protein expression levels of HK-2 cells under different LDHB inhibitor intervention times and found that Kim1 expression levels increased most significantly after 72 h of LDHB inhibitor intervention, while LDHB expression levels decreased significantly after 48 h of intervention and remained at a low level after 72 h (Figure 9A, Figure S5).

We further treated HK-2 cells with high glucose and 5 μM LDHB inhibitor, a concentration that induced morphological changes without altering cell viability. Through observation of the cellular cytoskeleton, we found that compared to the HG group, the F-actin microfilaments in the HG+LDHBi group were almost completely absent, with significantly reduced fluorescence intensity. The partially improved microfilament arrangement observed in the FBP1-overexpressed cells became structurally disorganized again after LDHB inhibitor intervention (Figure 9B).

In terms of glucose and energy metabolism, the elevated levels of cellular lactate and pyruvate corrected by FBP1 overexpression under high-glucose intervention further increased after concurrent use of the LDHB inhibitor (*p* < 0.05) (Figure 9C,D). Concurrent LDHB pharmacological inhibition attenuated the FBP1 overexpression-associated restoration of ATP and NAD+/NADH levels (*p* < 0.05) (Figure 9E,F).

In terms of mitochondrial damage, both mitochondrial-related protein expression and MitoTracker staining results showed that HK-2 cells suffered the most severe mitochondrial damage under the dual stress of high glucose and LDHB inhibition. The mitigation of mitochondrial injury by FBP1 overexpression was attenuated upon LDHB pharmacological inhibition (Figure 9G,H, Figure S6).

## 3. Discussion

The role of glucose metabolism in kidney disease has attracted increasing attention in recent years. The downregulation of glycolysis in proximal tubular epithelial cells leads to microvascular sparseness and fibrosis progression by inhibiting the metabolic reprogramming mediated by PFKFB3 [15]. Additionally, a recent study has confirmed that knocking out PC in mice increases mtDNA leakage, significantly activates the cGAS-STING pathway, and subsequently upregulates the expression of glycolytic enzymes such as HK2 and PKM2, promoting renal fibrosis. Therefore, PC may be a potential target for intervention in metabolic reprogramming and renal fibrosis [16]. A large number of molecules involved in the glycolysis pathway have been fully confirmed as potential targets for intervening in the progression of kidney diseases. Gluconeogenesis and glycolysis are oppositely regulated at most steps, yet studies on the role of gluconeogenesis in the progression of kidney diseases remain relatively scarce.

The kidneys account for 40% of endogenous gluconeogenesis, primarily occurring in the proximal tubules of the kidneys [17]. Hasegawa K et al. [18] observed downregulation of PCK1, a key enzyme in gluconeogenesis, in STZ-treated diabetic mice. They found that PCK1’s protective role in diabetic nephropathy is primarily achieved by maintaining mitochondrial ribosomal function and inhibiting apoptosis in proximal tubular cells. FBP1, a rate-limiting enzyme in the gluconeogenesis pathway, has been reported to suppress glycolytic flux and reduce lactate output in other tissue contexts. The absence of FBP1 is associated with increased expression of fibrosis-related genes in the liver [19], but its role in kidney disease, particularly in the progression of DKD, remains poorly understood. This study, using bioinformatics analysis, revealed that FBP1 expression is significantly downregulated in DKD patients and shows a significant positive correlation with eGFR, supporting FBP1 as a candidate molecular target worthy of further investigation in DKD pathophysiology.

The proximal tubule serves as the primary site of renal gluconeogenesis and is also the kidney cell with the highest energy demand [17]. In this study, we established renal tubular FBP1 overexpression and knockdown models via tail vein injection of AAV-9. The results showed that the degree of renal tubular atrophy was improved in the FBP1 overexpression group, while tubular atrophy was further exacerbated in the FBP1 knockdown group. Compared with the BKS-db group, mice in the knockdown group exhibited a significant decline in condition after 16 weeks of age, with markedly reduced spontaneous activity, impaired responsiveness, progressive weight loss, and significantly elevated serum creatinine and 24-h urine protein levels. We measured lactate, pyruvate, and lactate dehydrogenase activity in the kidneys and found that FBP1 overexpression reduced lactate and pyruvate levels. It is important to note that the current evidence supporting the FBP1–lactate–mitochondrial injury association is largely correlative and based on static endpoint measurements. While pharmacological LDHB inhibition suggests functional dependence, we cannot exclude off-target effects or nonspecific cytotoxicity, and direct evidence of gluconeogenic flux, lactate metabolic flux, or genetic rescue experiments is still lacking. All mechanistic interpretations in this study should be regarded as tentative hypotheses requiring further validation. Furthermore, the role of LDHB in mediating FBP1’s effects is inferred solely from pharmacological inhibition experiments. The lack of genetic LDHB knockdown/rescue models prevents definitive attribution of the observed phenotypes to LDHB loss-of-function. Direct metabolic flux tracing using isotope-labeled lactate or glucose substrates is also required to confirm whether FBP1 regulates mitochondrial function specifically through the lactate-pyruvate conversion step catalyzed by LDHB. At present, LDHB should be regarded as a candidate downstream effector rather than a confirmed mechanistic node.

As high-energy-consuming organs, the kidneys require significant energy to remove waste products from the blood, reabsorb nutrients, balance electrolytes and fluids, maintain acid-base homeostasis, and regulate blood pressure [20]. A balanced energy metabolism system is particularly important for maintaining the specific structure and physiological functions of the kidneys [21]. Mitochondria play a central role in energy metabolism within kidney cells. OMIC studies in DKD have demonstrated that mitochondrial dysfunction and the Warburg effect play key roles in its development [22,23]. In DKD patients, metabolites associated with mitochondrial function are reduced in urine, and levels of peroxisome proliferator-activated receptor gamma coactivator 1α (PGC1α), a major regulator of mitochondrial biogenesis, are decreased [22]. Decreased mitochondrial function is characterized by a shift from oxidative phosphorylation to glycolysis, leading to elevated lactate levels in urine and plasma [24]. In this study, we found that, compared with BKS-db group mice, urine lactate levels were significantly reduced in FBP1-overexpressing mice, whereas they were further elevated in FBP1-knockdown mice, suggesting decreased mitochondrial function and enhanced glycolytic activity. We observed that FBP1 overexpression was accompanied by upregulation of mitochondrial fusion proteins and downregulation of fission proteins in mouse kidneys, implying a potential link between FBP1 and mitochondrial quality regulation, though causal mechanisms remain to be defined.

To further clarify the mechanism of action of FBP1 in renal tubular epithelial cells, based on the results of this in vitro experiment, and given FBP1’s canonical role in gluconeogenesis, we speculate that the observed metabolic shifts may reflect altered flux through glucose metabolic pathways. However, this remains unproven without direct flux measurements. In the gluconeogenesis reaction using lactate as a substrate, the initial step is the conversion of lactate to pyruvate by LDHB. In this study, compared with the HG group, the FBP1 overexpression group showed a significant increase in LDHB expression and a significant decrease in cellular lactate content, suggesting that the initial reaction steps of the sugar anabolism process may be activated. However, compared with the HG group, the FBP1 overexpression group did not show an increase in renal pyruvate content but rather a significant decrease. We consider the following possibilities: the significant decrease in lactate content restored some mitochondrial function, allowing more pyruvate to enter the TCA cycle; the overexpression of FBP1 enhanced renal gluconeogenesis, effectively inhibiting glycolysis in renal tubular cells, thereby reducing pyruvate production. Notably, LDHA expression in the kidney tissue of DKD mice was increased compared with the WT Group, and LDHA expression changed in the opposite direction to FBP1. However, this effect was not observed in HK-2 cells treated with high glucose. We speculated that this might be due to overexpression of LDHA in proximal tubular epithelial cells, which led to a non-significant difference between groups.

NADH and NAD+ play multiple roles within cells, including energy metabolism, redox reactions, signal transduction, DNA repair, metabolic regulation, and cellular aging. They are critical for maintaining cellular function and metabolic balance. Previous studies have shown that diabetes increases the NADH/NAD+ ratio within cells, triggering reductive stress and subsequently affecting mitochondrial function. In this study, compared with the HG group, the NAD+/NADH ratio and ATP content were significantly elevated in the FBP1 overexpression group, suggesting improved mitochondrial function. We assessed mitochondrial morphology, mitochondrial fusion and fission protein expression, and membrane potential in HK-2 cells across all groups. Compared to the HG group, the FBP1 overexpression group exhibited more rounded mitochondrial morphology with more intact cristae, significantly increased fusion protein expression, and significantly decreased fission protein expression. We speculate that FBP1 may influence mitochondrial function by improving energy metabolism in renal tubular epithelial cells under high-glucose induction.

Cell lactate metabolism is closely related to LDH. The ratio of LDHA to LDHB subunits in the LDH tetramer determines the overall direction of the reaction. LDHB preferentially catalyzes the conversion of lactate to pyruvate in the renal tubular context studied here, serving as the initial step of lactate-dependent gluconeogenesis. By contrast, LDHA primarily drives pyruvate-to-lactate conversion under glycolytic conditions [25]. Research on the mechanisms of LDHB in kidney diseases is currently limited, and no studies have yet confirmed the association between LDHB and renal gluconeogenesis. In this study, we independently validated the adverse effects of LDHB inhibitors in vitro using HK-2 cells. Moderate concentrations of LDHB inhibitors significantly altered the microscopic morphology of HK-2 cells and elevated the expression of the renal tubular injury marker Kim1. These observations suggest that LDHB inhibition induces non-lethal cellular stress at 5 μM, which may confound the interpretation of phenotypic changes independent of FBP1 status.

FBP1 is a key enzyme in the gluconeogenic pathway, and the lactate-pyruvate axis serves as the initial step in gluconeogenesis, using lactate as a substrate; LDHB is an important regulatory enzyme in this metabolic axis. This study found that, under high-glucose induction, the levels of lactate and pyruvate, which were reduced by FBP1 overexpression, increased further upon treatment with an LDHB inhibitor, while ATP and NAD+/NADH ratios decreased further. Additionally, the mitochondrial damage induced by FBP1 overexpression was further exacerbated by LDHB inhibitors. Furthermore, based on the established correlation between FBP1 and LDHB in this study, and recent findings by Cuozzo et al. that LDHB helps improve lactate levels and basal insulin secretion in human pancreatic β-cells [26], this may partially explain why the FBP1 overexpression group exhibited mildly reduced blood glucose levels after 20 weeks compared to the DKD group, while blood glucose levels in the FBP1 knockdown group continued to rise. Based on the observed phenotypic associations between FBP1 and LDHB, we propose several plausible working hypotheses for future validation rather than definitive conclusions: ① Via the NF-κB signaling pathway: Wencheng Zhu et al. [27] found in their study using molecular modeling to predict new functions of metabolic enzymes that FBP1 can dephosphorylate IκBα, thereby inhibiting NF-κB signaling activation and reducing the release of inflammatory and oncogenic factors. The NF-κB pathway is known to regulate the expression of various metabolic enzymes and may influence LDHB through downstream targets. ② Accumulation of acetyl-CoA: Reduced FBP1 activity may impair gluconeogenesis, causing cells to rely on fatty acid oxidation, which generates large amounts of acetyl-CoA. Acetyl-CoA can inhibit pyruvate dehydrogenase, leading to increased conversion of pyruvate to lactate. The accumulation of lactate may upregulate LDHB expression to adapt to metabolic demands. ③ Feedback from the lactate cycle: FBP1-mediated gluconeogenesis and lactate metabolism involving LDHB may form a dynamic equilibrium. For example, when lactate serves as a substrate for gluconeogenesis, its accumulation may regulate LDHB expression through pH changes or metabolic feedback mechanisms. It is important to emphasize that these three mechanisms are currently speculative and require rigorous validation through genetic rescue experiments or targeted signaling assays.

This study also has some limitations. Firstly, in the in vivo experimental section, we selected tail vein injection of AAV-9 to construct FBP1 overexpression and knockdown models. Although we selected the Ksp-cadherin promoter to target proximal tubules, compared with specific gene knockout mice, AAV has poor tissue specificity. Some promoter activities may not be entirely precise, leading to off-target effects or uneven expression levels. Additionally, during long-term expression, AAV may undergo methylation modifications or chromatin compaction, resulting in a gradual decline in transcriptional activity, which may affect the construction of chronic disease models. Although we employed a two-injection approach in this study, to more precisely describe the role of FBP1 in DKD tubular damage, further studies using specific gene-edited mice are required. It is important to note that although we employed the Ksp-cadherin promoter to target renal tubules, we did not experimentally validate the tubular specificity of AAV9-mediated transduction in this study. Systemic delivery of AAV9 may result in off-target expression in other highly perfused organs, such as the liver or heart. Consequently, we cannot exclude the possibility that some of the observed systemic metabolic effects (e.g., blood glucose trends) or renal phenotypes may be influenced by extra-tubular FBP1 expression. Future studies utilizing tubular-specific Cre-loxP systems or direct quantification of vector biodistribution are required to definitively confirm the cellular specificity of our findings. Furthermore, LDH expression levels in tissue and cell samples do not equate to LDH expression levels in blood supernatants. To better apply LDH as a clinical indicator, we believe further research is needed to clarify the release pathways of LDH from kidney and renal tubular epithelial cells and their correlation with serum LDH expression levels. Secondly, in the animal experiment section, we found significant differences in LDHA expression levels among the normal group, DKD group, and FBP1 overexpression and knockdown groups. However, due to extremely high LDHA expression levels in the cell experiment section, no significant differences were observed, and no further studies on LDHA were conducted. This may be because HK-2 cells are immortal cell lines that alter basal metabolic rates, and the role of LDHA in energy metabolism in renal tubular epithelial cells cannot be overlooked. Further research is still needed to explore and refine the mechanisms underlying the lactate-pyruvate metabolic axis in DKD. Third, the current evidence supporting the FBP1-LDHB axis is largely correlative and based on static endpoint measurements. While pharmacological LDHB inhibition suggests functional dependence, we cannot exclude off-target effects or nonspecific cytotoxicity, and direct evidence of gluconeogenic flux, lactate metabolic flux, or genetic rescue experiments is still lacking. All mechanistic interpretations in this study should be regarded as tentative hypotheses requiring further validation. Fourth, the role of LDHB in mediating FBP1’s effects is inferred solely from pharmacological inhibition experiments. The lack of genetic LDHB knockdown/rescue models prevents definitive attribution of the observed phenotypes to LDHB loss-of-function. Thus, LDHB should be regarded as a candidate downstream effector rather than a confirmed mechanistic node. Fifth, this study infers enhanced renal gluconeogenesis solely from FBP1 expression changes and static metabolite measurements. No direct evidence of glucose production, gluconeogenic flux, or substrate tracing is provided. Reduced lactate/pyruvate levels may stem from multiple alternative mechanisms, including altered glycolysis, mitochondrial oxidation, or generalized metabolic stress. Thus, activation of renal gluconeogenesis is a speculative interpretation rather than a demonstrated finding.

## 4. Materials and Methods

### 4.1. Microarray Data Processing and Identification of Genes Related to Glucose Metabolism

Two DKD renal tubule microarray datasets were collected from the GEO database. These were GSE30529 (GPL571) [28], which included 10 DKD patients and 12 control samples, and GSE104954 (GPL22945) [29], which included 7 DKD patients and 18 control samples. Batch effects between GSE30529 and GSE104954 were removed using the ComBatfunction of the svaR package. Principal component analysis confirmed that batch variation was eliminated prior to differential expression analysis.

Gene sets associated with glucose metabolism were retrieved from the Molecular Signatures Database (MSigDB) of the Gene Set Enrichment Analysis (GSEA) project, including HALLMARK_GLYCOLYSIS, KEGG_GLYCOLYSIS_GLUCONEOGENESIS, and REACTOME_GLYCOLYSIS (http://software.broadinstitute.org/gsea/index.jsp (accessed on 29 November 2024)). After removing overlapping genes, a total of 290 genes related to glucose metabolism were obtained.

### 4.2. Functional Enrichment Analysis and Screening of Core Genes Related to Glucose Metabolism

The gene co-expression network was constructed using the R package called “WGCNA”. (version 1.73) The adjacency matrix was composed of weighted correlation coefficients. Subsequently, the adjacency matrix was converted into a topological overlap matrix (TOM). Soft threshold power 10 and minimum module size 300 were set to screen for core modules. Modules were then tested using Pearson correlation analysis with a significance threshold of *p* < 0.05. To identify differentially expressed genes (DEGs) between DKD and healthy samples, the limma R software package (version 3.64.3) was used. The cutoff criteria were adjusted to *p* < 0.05 and log|(FC)| > 0.8. The ggplot2 package (version 4.0.1) was used to visualize the distribution of differentially expressed genes using a volcano plot. We intersected glucose metabolism-related genes with DKD-related module genes from WGCNA and DKD differentially expressed genes. A Venn diagram was used to describe the details of the overlapping genes. Next, a least absolute shrinkage and selection operator (LASSO) regression model was built to determine the optimal value of λ and construct a core candidate genes. The LASSO algorithm was used for variable selection and shrinkage based on the “glmnet” R package (version 4.1.10).

### 4.3. Clinical Data Validation and Diagnostic Efficacy of Hub Genes in DKD Assessment

To identify glucose metabolism-related hub genes, we queried the Nephroseq v5 platform (http://v5.nephroseq.org) and performed comparative analysis of mRNA expression profiles of core candidate genes in DKD tissues versus healthy control samples. No original human tissue samples were prospectively collected or analyzed in this study.

### 4.4. Reagents

Glucose, pyruvate, and L-LDH activity assay kits were purchased from BOXbio (Beijing, China, #AKSU001M, #AKAC002M, #AKCO003M). The lactate assay kit was purchased from Elabscience (Wuhan, China, #E-BC-K044-M). The creatinine assay kit was purchased from Rayto (Shenzhen, China, #S03076). The BCA assay kit was obtained from Beyotime Biotechnology (Shanghai, China, #P0010). DMEM medium and fetal bovine serum were purchased from Gibco (Grand Island, NY, USA; #A5256701). PBS and electron microscopy fixative were purchased from Servicebio (Wuhan, China, #G4202). RIPA lysis buffer and cocktail were purchased from Beyotime Biotechnology (Shanghai, China). All cell culture plates were purchased from NEST Biotechnology (Wuxi, China). LDHB inhibitor was purchased from MedChemExpress (Monmouth Junction, NJ, USA, #HY147216). SDS-PAGE protein loading buffer was obtained from Affinibody LifeScience AG (Liestal, Switzerland). MitoTracker and Hoechst staining reagents were purchased from Meilunbio (Dalian, China). Phalloidin F-actin dye was obtained from Immunoway (Plano, TX, USA). Glucose powder was purchased from BioFRoxx (Einhausen, Germany), and mannitol powder was obtained from HUSHI (Shanghai, China).

The primary antibodies were obtained as follows: FBP1, OPA1, Fis were provided by ThermoFisher Scientific (Waltham, MA, USA, #MA5-35149, #MA5-32786, #MA5-35336), Mitofusin-2 was from Cell Signaling (Danvers, MA, USA, #9482S), Drp1 was obtained from Abcam (Cambridge, UK, #ab184247), LDHA and LDHB were obtained from Proteintech (Wuhan, China, #19987-1-AP, #19988-1-AP), Kim1 was purchased from Sigma Aldrich (St. Louis, MO, USA, #ABF199), and HRP Goat Anti Mouse/Rabbit IgG(H+L)-S was purchased from immunoway (Plano, TX, USA, #RS0011).

### 4.5. Animal Experiment

In this study, 8-week-old BKS-db (ko/ko) and BKS/m mice were purchased from Guangdong Jituo Pharmaceutical Biotechnology Co., Ltd. (Guangzhou, China). The mice were housed at the Experimental Animal Center of Fujian Medical University. All procedures involving laboratory animals were conducted in accordance with the approved protocol of the Animal Research Committee of Fujian Medical University (IACUC FJMU2023-Y-1033, 28 July 2023) and complied with the “Guidelines for the Care and Use of Laboratory Animals.”

Sample size was calculated using G*Power 3.1 based on pilot data showing a 30% difference in 24-h urinary protein levels between BKS-db and WT mice (α = 0.05, power = 0.8), yielding a minimum of 6 mice per group. We enrolled 8 mice per group to account for potential attrition, in line with the 3R principle. Exclusion criteria were predefined in the IACUC-approved protocol: death prior to endpoint, failure to establish hyperglycemia (fasting glucose < 16.7 mmol/L at 10 weeks of age), or severe infection. No animals were excluded from the final analysis. Mice were housed 4–5 per cage under specific pathogen-free conditions (12-h light/dark cycle, 22 ± 2 °C, 50 ± 10% humidity), with ad libitum access to standard chow and water. Humane endpoints included >20% body weight loss, severe lethargy, or inability to access food/water; no animals reached these endpoints. Cage allocation was randomized using a computer-generated sequence, and all cages were rotated weekly to minimize environmental bias.

Body weight and fasting blood glucose were monitored every 4 weeks. At the end of the intervention, 24-h urine was collected and albumin excretion was monitored. Mice were euthanized, and kidneys were removed. Kidney samples were fixed overnight in 4% paraformaldehyde or stored at −80 °C.

Investigators performing phenotyping, tissue collection, and data analysis were blinded to group allocation throughout the experiment. Urine collection, euthanasia, and tissue processing were performed with coded samples to prevent unblinding.

### 4.6. Adeno-Associated Virus-9 (AAV-9) Infected Mice

**Figure 10:**
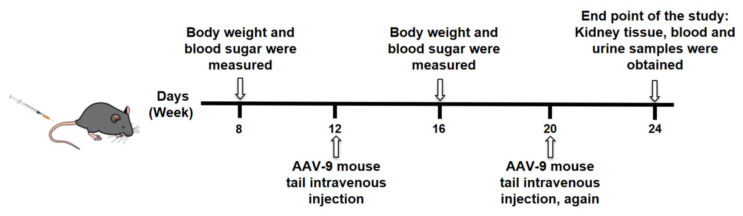
Construction of Experimental Animal Models and Experimental Protocols.

To overexpress and knockdown FBP1 in the kidneys, BKS-db mice were injected with AAV-9 into the tail vein. AAV-9 was provided by Shanghai Genechem Co., Ltd. (Shanghai, China). To ensure efficient overexpression or knockdown, we administered two injections of AAV-9, with detailed experimental procedures outlined in Figure 10. The injection doses were 1 × 10^12^ vector genome (vg)/mL and 7.5 × 10^11^ vector genome (vg)/mL, respectively.

### 4.7. Histological and Immunohistochemistry Analysis

Kidney tissues were fixed in 4% paraformaldehyde, embedded in paraffin, and sectioned at 5 μm. Sections were deparaffinized, rehydrated through a graded ethanol series, and subjected to antigen retrieval by microwave heating in citrate buffer (pH 6.0). Endogenous peroxidase activity was blocked with 3% hydrogen peroxide, followed by blocking with 3% bovine serum albumin.

Sections were incubated overnight at 4 °C with a recombinant rabbit monoclonal anti-FBP1 antibody (clone 7N8C1, Catalog# MA5-35149; Thermo Fisher Scientific, Waltham, MA, USA) at a dilution of 1:100, as validated by the manufacturer for immunohistochemistry on paraffin-embedded kidney tissues. The antibody was raised against a synthetic peptide corresponding to amino acids 239–338 of human FBP1 (UniProt ID: P09467) and purified by affinity chromatography. According to the manufacturer’s technical datasheet, this antibody exhibits specific cytoplasmic staining in renal tubular epithelial cells of mouse and rat kidney tissues.

After washing, sections were incubated with a horseradish peroxidase-conjugated secondary antibody for 50 min at room temperature. Immunoreactivity was visualized using diaminobenzidine (DAB) substrate, and nuclei were counterstained with hematoxylin.

For quantitative analysis of FBP1 immunohistochemical staining, three random non-overlapping fields of renal cortex were captured per sample at ×400 magnification using consistent acquisition parameters. Integrated optical density (IOD) of positive tubular epithelial staining was measured using Image J software (National Institutes of Health, Bethesda, MD, USA, 1.51d), and the average IOD across the three fields was used to represent relative FBP1 expression.

### 4.8. Cell Culture and Treatment

In the cell experiments, all data were obtained from at least three independent biological replicate experiments, with each experiment containing three technical replicates. HK-2 cells were purchased from Procell Biotechnology Co., Ltd. (Wuhan, China, #CL-0109) (STR-verified) and cultured at 37 °C in a 5% CO_2_ atmosphere in low-glucose DMEM containing 10% fetal bovine serum. FBP1 overexpression and knockdown models were constructed using lentiviruses provided by Shanghai Genechem Co., Ltd. (Shanghai, China). High-glucose and mannitol powders were added to pre-prepared HK2 culture medium at appropriate concentrations to formulate high-glucose or mannitol culture medium with a final concentration of 30 mmol/L for cellular intervention. Different concentrations of LDHB inhibitors were used to treat HK-2 cells for varying lengths of time to determine the optimal treatment duration and concentration. Ultimately, 5μM LDHB inhibitor treatment for 72 h was selected for subsequent experiments.

### 4.9. Western Blots

Lysate kidney tissue or cells in RIPA lysis buffer on ice, then centrifuge at 13,000× *g* for 15 min at 4 °C. Collect the supernatant and measure it using the BCA assay kit. Separate equal amounts of protein from SDS-PAGE and transfer them to an NC membrane (Millipore Corporation, Billerica, MA, USA). Then, incubate the samples with the primary antibody at 4 °C overnight, followed by staining with HRP-conjugated secondary antibody. Quantify the bands using Image J software. The statistical column chart of the cell experiment part is in the Supplementary Materials.

### 4.10. Analysis of Glucose, Lactic Acid, Pyruvic Acid, and Lactate Dehydrogenase Activity

Frozen kidney tissue or cells are homogenized on ice with PBS, then centrifuged at 12,000× *g* for 15 min at 4 °C. The supernatant is collected and tested using a lactate colorimetric assay kit according to the manufacturer’s instructions.

### 4.11. ATP Content Detection

Aspirate the culture medium and lyse the cells at a ratio of 200 μL of lysis buffer per well in a 6-well plate. After lysis, centrifuge at 12,000× *g* at 4 °C for 5 min, collect the supernatant, and proceed with testing according to the manufacturer’s instructions.

### 4.12. NAD+ and NADH Content Detection

Aspirate the culture medium. Using a pipette, add 200 μL of NAD+/NADH extraction solution at a ratio of 200 μL per 1 million cells, and gently tap to promote cell lysis. Centrifuge at 12,000× *g* at 4 °C for 5–10 min, collect the supernatant, and proceed with testing according to the manufacturer’s instructions.

### 4.13. Electron Microscopic Observation of Mitochondrial Morphology

For tissue samples, fresh renal cortex was dissected into 1 mm^3^ fragments and immediately fixed in 2.5% glutaraldehyde at 4 °C for 24 h. Samples were sequentially dehydrated in graded ethanol, embedded in epoxy resin, and sectioned into 50-nm ultrathin sections using an ultramicrotome (Leica EM UC7, Leica Microsystems GmbH, Wetzlar, Germany). Sections were counterstained with 1% uranyl acetate for 20 min and 2% lead citrate for 10 min, then examined under a transmission electron microscope (Hitachi HT7700/80 kV, Hitachi High-Tech Corporation, Tokyo, Japan) to evaluate mitochondrial ultrastructure.

For cell samples, HK-2 cells cultured in 6-well plates were washed twice with pre-warmed PBS, fixed with 2.5% glutaraldehyde at room temperature for 5 min, and detached using a cell scraper. Cell suspensions were collected by centrifugation at 2000× *g* for 2 min, resuspended in fresh fixative, and processed following the same embedding, sectioning, and staining procedures as tissue samples.

### 4.14. Observation of Mitochondrial Network Morphology Using MitoTracker

MitoTracker dye was added to the culture medium, and cells were cultured at 37 °C for 30 min. Cells were gently washed, fixed, and double-stained with Hoechst to visualize cell nuclei. Images were then captured using a confocal microscope (LSM 800; Carl Zeiss Microscopy GmbH, Jena, Germany).

### 4.15. Cytoskeletal Staining

Place autoclave-treated cell smears in a sterile 12-well cell culture plate, fix with 4% paraformaldehyde, break the membrane with 0.5% Triton X-100 at room temperature, block with 1% BSA at room temperature for 30 min, add F-actin dye phalloidin, stain with DAPI, cover the slide, and observe and collect images using a confocal microscope.

### 4.16. Statistical Analysis

Prior to statistical analysis, the normality of the data was assessed using the Shapiro–Wilk test, and the homogeneity of variances was evaluated using Levene’s test. For data meeting parametric assumptions, comparisons between two groups were performed using Student’s *t*-test, and comparisons among multiple groups were performed using one-way analysis of variance (ANOVA) followed by Tukey’s post hoc test. If the data violated the assumptions of normality or homogeneity of variances, non-parametric methods were employed, such as the Mann–Whitney U test for two-group comparisons or the Kruskal–Wallis test for multiple-group comparisons. In addition, body weight and blood glucose were measured serially over time; however, due to the current analytical scope, statistical comparisons were performed independently at each scheduled time point. Data are presented as mean ± standard deviation, and all analyses were conducted using GraphPad Prism software (version 10.0). *p*-value < 0.05 was considered statistically significant.

## 5. Conclusions

**Figure 11:**
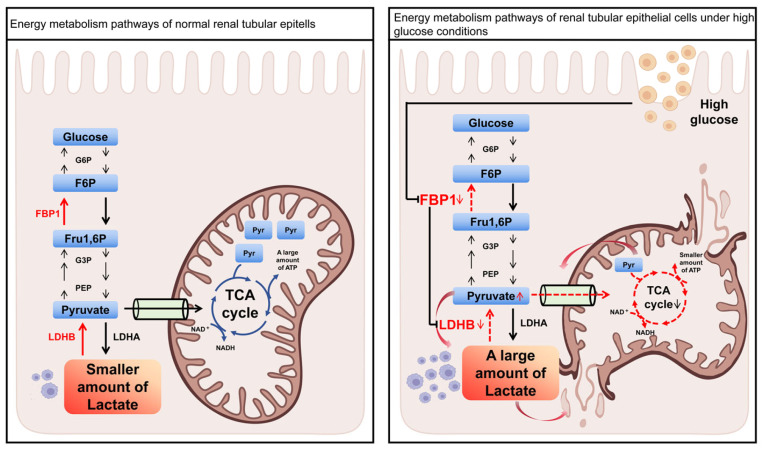
Mechanism diagram: During the progression of DKD, FBP1 expression decreases significantly and renal gluconeogenesis capacity decreases, leading to a decrease in LDHB expression, which is necessary for the conversion of lactate to pyruvate. Lactate accumulates in the kidneys, and excessive lactate causes damage to the mitochondria of renal tubular epithelial cells. Abbreviations: FBP1 (Fructose-1,6-bisphosphatase 1), G6P (Glucose-6-phosphate), F6P (Fructose-6-phosphate), Fru1,6P (Fructose-1,6-bisphosphate), G3P (Glyceraldehyde-3-phosphate), PEP (Phosphoenolpyruvate), Pyr/Pyruvate (Pyruvate), LDHB (Lactate Dehydrogenase B), LDHA (Lactate Dehydrogenase A), TCA cycle (Tricarboxylic Acid Cycle), NAD^+^ (Nicotinamide Adenine Dinucleotide, oxidized form), NADH (Nicotinamide Adenine Dinucleotide, reduced form), ATP (Adenosine Triphosphate).

In summary, this study observed that FBP1 expression is significantly decreased during DKD progression, which correlates with reduced LDHB expression and renal lactate accumulation. These changes are concurrently associated with mitochondrial damage in renal tubular epithelial cells. Whether these phenotypic associations are causally linked to altered renal gluconeogenesis requires direct validation in future studies (Figure 11).

## Data Availability

The original contributions presented in this study are included in the article. Further inquiries can be directed to the corresponding authors.

## Supplementary Materials

The following supporting information can be downloaded at: https://www.mdpi.com/article/10.3390/ijms27135906/s1.

## Abbreviations

The following abbreviations are used in this manuscript:

AAV-9	Adeno-associated virus serotype 9
DEGs	Differentially expressed genes
DKD	Diabetic kidney disease
ESRD	End-stage renal disease
eGFR	Estimated glomerular filtration rate
FAO	Fatty Acid Oxidation
FBP1	Fructose-1,6-bisphosphatase 1
GSEA	Gene Set Enrichment Analysis
HK-2	Human renal tubular epithelial
LASSO	Least absolute shrinkage and selection operator
LDHB	Lactate Dehydrogenase B
Oxphos	Oxidative phosphorylation
TCA	Tricarboxylic Acid
WGCNA	Weighted Gene Co-expression Network Analysis
