# Supplementary figures and images for: FBP1 Is Associated with Attenuated Mitochondrial Injury in Renal Tubular Epithelial Cells of Diabetic Kidney Disease via Modulation of Lactate Metabolism
Source: Int J Mol Sci. 2026 Jun 30;27(13):5906. doi: 10.3390/ijms27135906 (PMC13361246; doi:10.3390/ijms27135906)

Figure S1

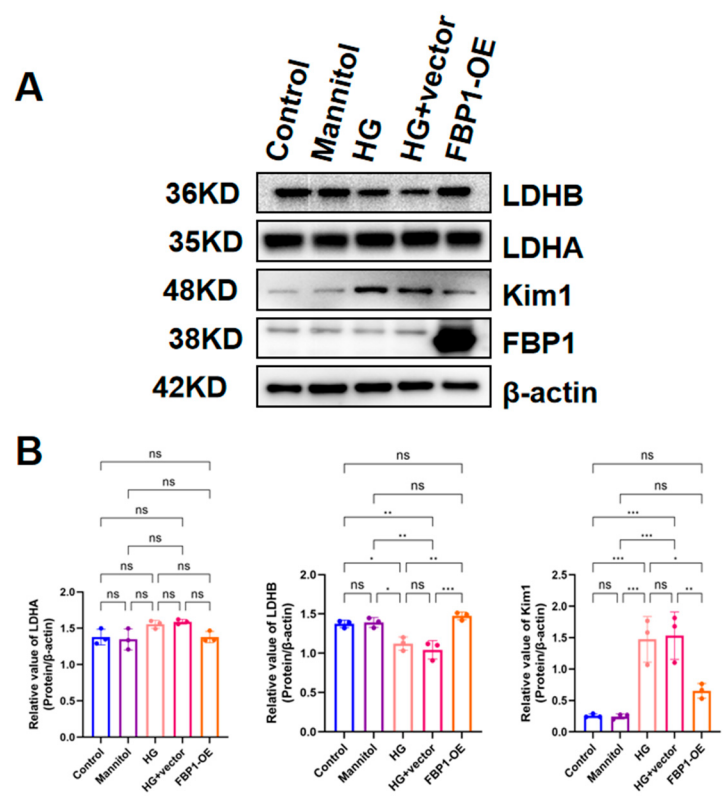

Figure S2

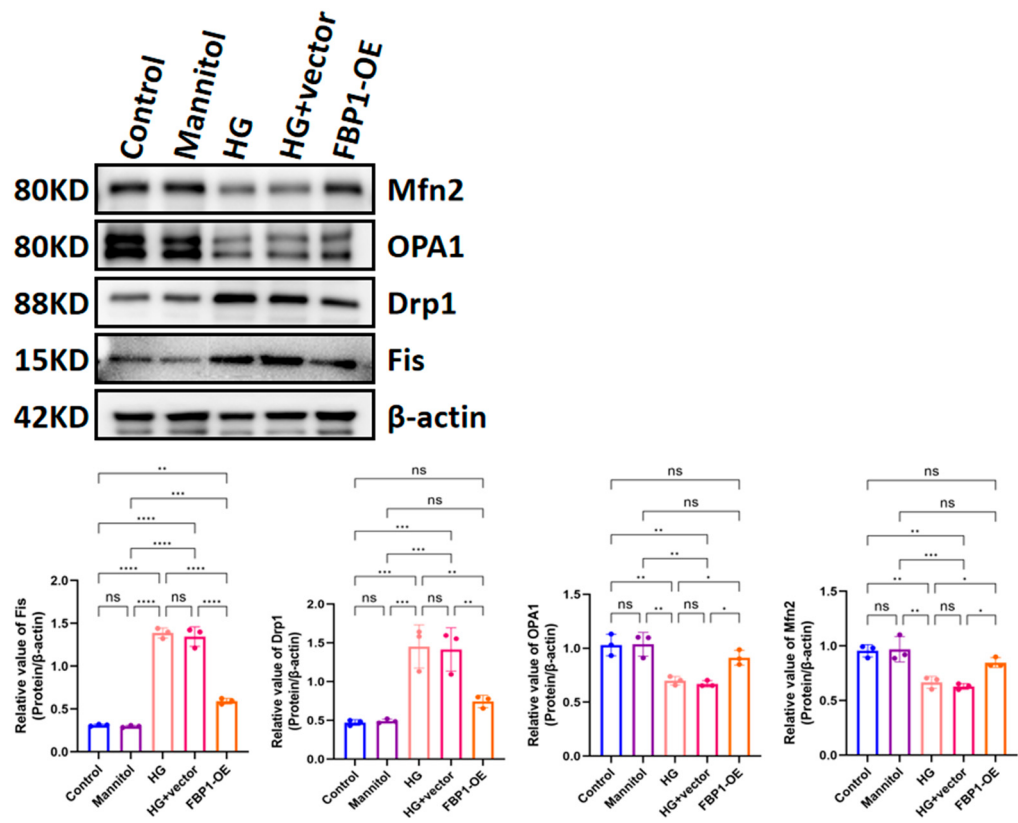

Figure S3

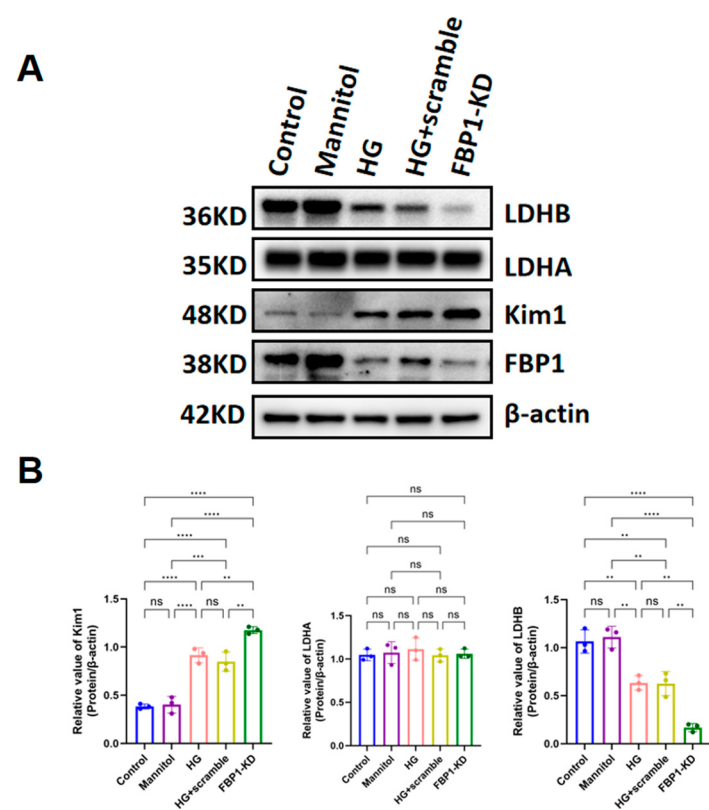

Figure S4

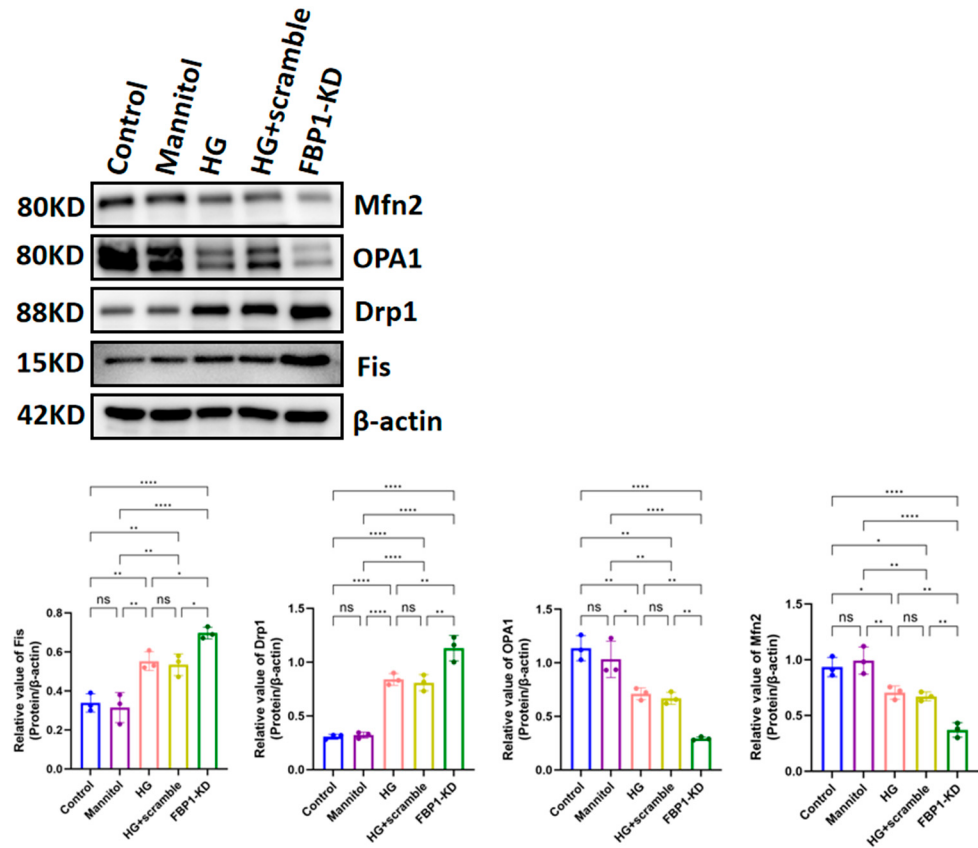

Figure S5

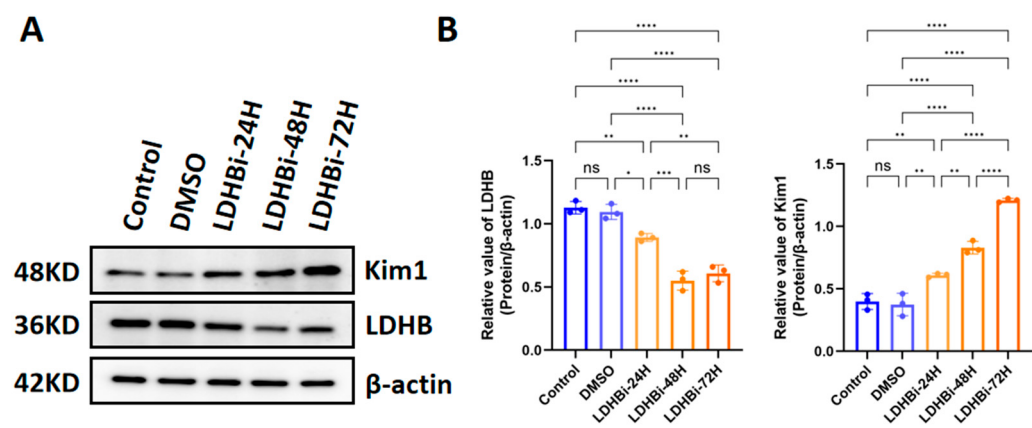

Figure S6

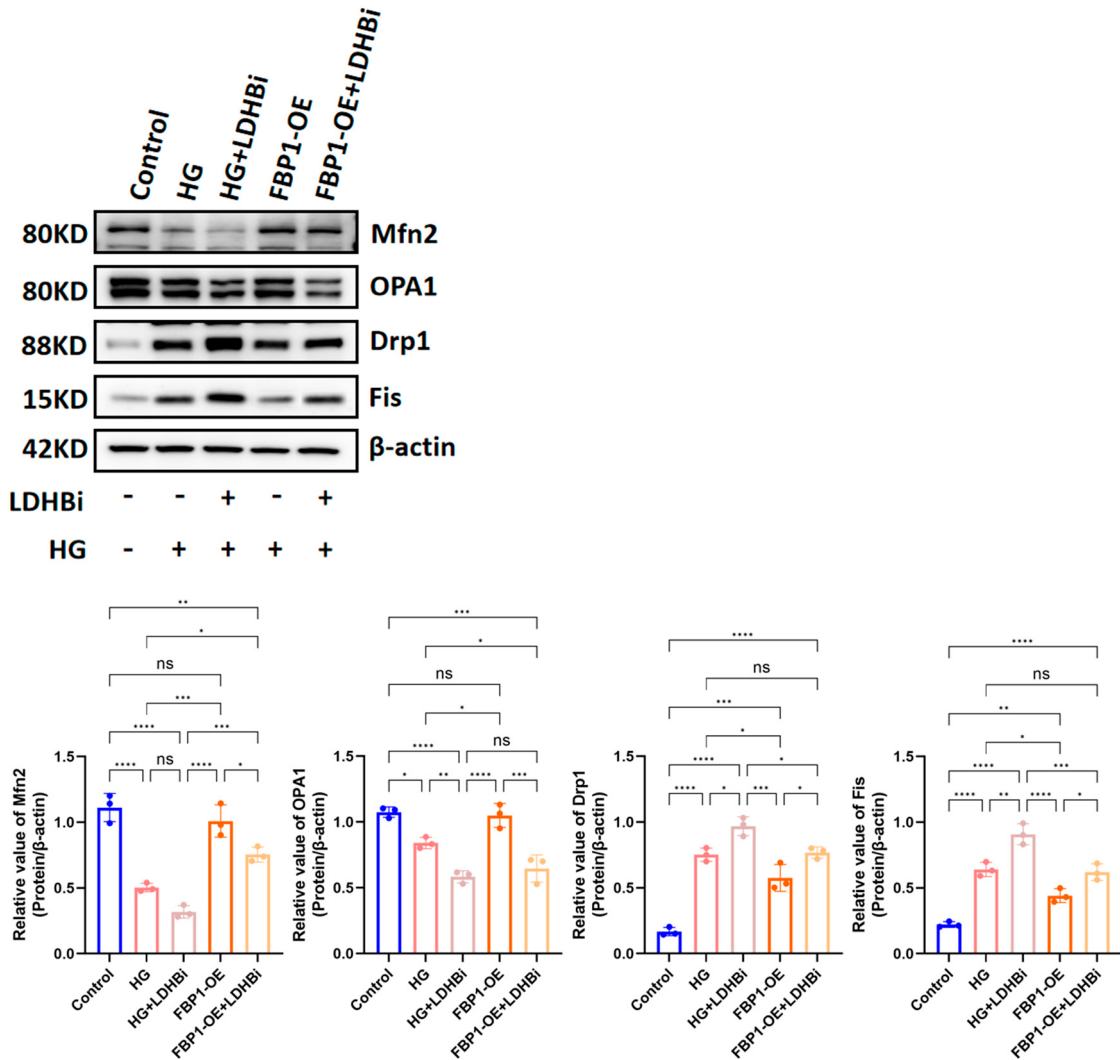

Supplement: Supplementary file 1 [file ijms-27-05906-s001.zip › ijms-4255511-supplementary.pdf]
